# Emulsifiers from Partially Composted Olive Waste

**DOI:** 10.3390/foods8070271

**Published:** 2019-07-20

**Authors:** Aikaterini Koliastasi, Vasiliki Kompothekra, Charilaos Giotis, Antonis K. Moustakas, Efstathia P. Skotti, Argyrios Gerakis, Eleni Kalogianni, Christos Ritzoulis

**Affiliations:** 1 Department of Food Science and Technology, International Hellenic University, Sindos Campus, 57400 Thessaloniki, Greece; 2 Department of Food Science and Technology, Ionian University, Vergoti Avenue, 28100 Argostoli, Greece

**Keywords:** emulsifier, olive waste, size exclusion chromatography (SEC), emulsion, compost, Ostwald ripening

## Abstract

Partial (one month) composting of solid olive processing waste is shown to produce extractable emulsifiers. Size exclusion chromatography (SEC) and Fourier-transform infra-red spectroscopy (FTIR) show that these consist of polysaccharides and proteins from the composted waste. Aqueous extraction at pH 5, pH 7, and pH 9 all yield extracts rich in oligosacchrides and oligopeptides which derive from the break-down of the macromolecules under composting, with the extract obtained at pH 5 being the richer in such components. Fourier-transform infra-red (FTIR) spectroscopy also confirms that these materials consist of proteinic and poly/oligosaccharidic populations. These materials can emulsify stable oil–in–water emulsions at pH 3 for a few days, while the same emulsions collapse in less than 24 h at pH 7. Confocal microscopy and droplet size distribution data suggest that Ostwald ripening, rather than coalescence, is the major course of emulsion instability. The above point to a short-process alternative to full composting in producing a high added value product from solid olive processing waste.

## 1. Introduction

Olive oil is produced mainly in the Mediterranean area and EU countries with the average olive oil production in the EU in recent years being 2.2 million tons, representing around 80% of world production with half of it being produced in Spain [1]. Greece, Italy, and Spain account for about 97% of EU olive oil production [2]. Although olive oil has a positive effect on our health, its resulting by-products (olive mill waste) are acknowledged as a serious environmental threat, especially in the aforementioned countries [3]. The characteristics of olive mill waste vary, depending on factors such as the method of extraction, variety, and maturity of olives, region of origin, climatic conditions, and associated cultivation/processing methods [4]. The disposal of these wastes is very crucial because olive mill waste has been shown to affect the physical and chemical properties of soil and its microbial community, while several studies have evidenced its phytotoxic effects and antimicrobial activity. Olive oil waste water can be toxictoanaerobic bacteria, which may in hibit conventional secondary and anaerobic treatments in municipal treatment plants [5]. Among the possible technologies for recycling the two-phase olive-mill wastewater (TPOMW), composting is one of the most promising options to transform this material into a valuable organic commodity [6]. Composting is a bio-chemical aerobic degradation process of organic waste materials. Under suitable conditions, composting has three consecutive phases: (a) An initial activation phase, (b) a thermophilic phase recognized by a sudden temperature increase, and (c) a mesophilic phase where the organic materials cool down to the surrounding temperature [7]. Microbial metabolic activities generate heat, which leads to physicochemical changes of the organic matter into biomass, CO_2_, and humus-like end-products; at the end of the process, a stable, humus-rich, complex mixture is produced [8]. This process leads to the formation of materials that are not associated with the microbiota inhibition and phytotoxicity, which relate to the direct application of the untreated wastes onto the soil [6].

Our group has previously extracted and characterized emulsifiers from food processing waste, e.g., from quince seed [9], winery waste [10], and olive mill waste [11]. In a recent work of ours [12], olive compost has been shown to produce interfacially-active materials with substantial emulsifying capacity. The emulsifiers obtained after composting olive mill waste showed better emulsifying and stabilizing properties compared to the ones produced from uncomposted waste. However, the full composting process cycle is time consuming (6 months). Waste processing is a very low-return operation, so it is crucial to restrict its timescale. As non-composted olive processing waste extracts have some emulsifying capacity [11], it is worthy to investigate whether partial composting is sufficient to increase the emulsifying capacity in a restricted amount of time, e.g., with one month’s treatment, rather than the full six-months operation. To this end, this work investigates the capacity of olive processing waste to produce, after a short (1-month) accelerated composting, extracts with enhanced emulsifying capacity.

## 2. Materials and Methods

### 2.1. Materials

Tris (hydroxymethyl)-aminomethane, sodium phosphate dihydrate and trihydrate, sodium hydroxide, and acetic acid were purchased from Merck (Darmstand, Germany). Petroleum ether was obtained from Sigma (St. Louis, MO, USA) and hydrochloric acid from Chem-lab NV (Zedelgem, Belgium). Miglyol812 N (Cremer Oleo GmbH & Co., Hamburg, Germany) was used as the oil phase for the interfacial measurements. Extracts were filtered by Whatman filter papers (125mm). Ultrapure water (18.2 MΩ) was obtained from an Ultraclear Ro DI 30 device (Evoqua Lab, Pittsburgh, PA, USA).

### 2.2. Composting

Olive mill waste (79% *w*/*w* moisture) on 1.4 m^3^ was collected from a two-phase organic olive mill in Kefalonia, Greece. After 2 days of ambient drying, 0.7 m^3^ of fresh olive leaves and 10 kg of NH_4_NO_3_ fertilizer (Nutrammon, Hellagrolip SA, Athens, Greece) were added with the waste into a purpose-built pit as layers of raw waste, olive leaves, and fertilizer. The compost pile was covered with a plastic sheet to protect it from winter rains. The sheet was removed between storms to promote aeration. The compost core temperature was recorded weekly. Following the beginning of the aerobic fermentation stage, the compost pile was bi-weekly overturned by transfer into an adjacent pit. 

### 2.3. Extraction

The 1-month compost was placed in a vacuum oven (90 °C) until it dried and then it underwent Soxhlet extraction of its lipids with petroleum ether. 10 g of this sample were extracted with either: 0.05 M sodium acetate buffer at pH 5 at 70 °C for 30 min (from now sample OC5), or 0.05 M phosphate buffer at pH 7 at 70 °C for 30 min (from now sample OC7), or 0.05 M tris buffer pH 9 at 70 °C for 30 min (from now sample OC9). The extractions were held in parallel (i.e., each defatted sample was extracted at one pH value (at pH 5, 7, or 9), while extractions at a single pH value were performed sequentially for 3 consecutive times, using 100mL buffer per run. The samples were then centrifuged for 25 min at 3500 rpm in order to separate the extracted matter from the insoluble residue. The supernatants were filtered out and then lyophilized and stored for further use. The materials were reconstituted for use by dissolving in an appropriate volume of de-ionized water under magnetic stirring. They were then enclosed in a dialysis membrane (3500 MWCO) and were immersed in ultrapure water. The water was renewed thrice per day over 2 days. The dialysis membrane content was lyophilized and stored. 

### 2.4. Emulsion Preparation

One of either OC5, OC7, or OC9 (see Section 2.3) were added into buffers of 10 mM Trizma buffer and 1 mM sodium azide set at pH 7 as to prepare 8 mg mL^−1^ solutions. These were magnetically stirred as to dissolve the extracts and were then mixed with miglyol acting as a model oil phase (oil volume fraction *φ* = 0.1). These two phases (buffered solution of extracts and miglyol) were magnetically stirred as to prepare a crude pre-mix. This pre-mix was treated with a laboratory ultrasonic homogenizer (Hielscher UP-100H, Teltow, Germany) for a duration of 30 s of continuous treatment. For their long-term monitoring, each emulsion was transferred into a sealed tube and was stored at ambient temperature under dark and quiescent conditions.

### 2.5. Size Exclusion Chromatography (SEC)

Size exclusion chromatographs were obtained using a setup made of (i) a SpectraSystem SCM 1000 degasser (Thermo Separation Products, San Jose, CA, USA), followed in a series by (ii) a SpectraSystem P 2000 chromatographic pump (Thermo Separation Products, San Jose, CA, USA), then (iii) a column system comprising of a 2 μm frit (Idex, Oak Harbor, WA, USA), then (iv) a GPC/SEC PL-Aquagel-OH 50 × 7.5 mm 8 μm guard column (Varian Inc., Palo Alto, CA, USA), then (v) two GPC/SEC PL-Aquagel-OH 300 × 7.5 mm columns (Varian Inc.) placed in a Model 605 column oven (Scientific Systems Incorporated, State College, PA, USA) set at 30 °C, then (vi) a UV detector recording absorbance at 280 nm (Rigas Labs, Thessaloniki, Greece), and (vii) a BI-MwA laser light scattering detector (MALLS) (Brookhaven Instruments Corporation, Brookhaven, Holtsville, NY, USA). Recording and treating results were handled with ParSec, a dedicated software (ParSec, Brookhaven Instruments Corporation, Brookhaven, Holtsville, NY, USA). Measurements were carried by means of filtering using a 1 μm syringe filter, followed by injecting 200 μL of 8 mg mL^−1^ sample at a flow rate of 0.8 mL min^−1^. The reader should be reminded here that SEC lets elute the larger-sized molecules first, then followed by molecules of smaller sizes, ending with the smallest of molecules.

### 2.6. Fourier-Transform Infra-Red Spectroscopy (FTIR)

Fourier transform infra-red (FTIR) spectra of the solid samples were recorded using the Attenuated Total Reflection (ATR) Smart Orbit diamond reflection accessory (Thermo Electron Corporation, Madison, WI, USA) of a Thermo Nicolet 380 IR spectrometer (Thermo Electron Corporation). 

### 2.7. Measurements of Droplet Distribution

The droplet size distributions of miglyol-in-water emulsions emulsified by the materials under study were measured a Malvern Mastersizer 2000 (Malvern Instrument, Malvern, Worcesteshire, UK) apparatus operating with a Hydro MU liquid sampler (Malvern Instrument). The results were treated using the Mie scattering model, assuming spherical particles, a continuous phase diffraction index of 1.33, a dispersed phase refractive index of 1.42, and an absorbance value of 0.1. 

### 2.8. Zeta Potential Measurements

Zeta potential values were obtained with a Brookhaven ZetaPALS apparatus (Brookhaven Instruments Corporation, Brookhaven, Holtsville, NY, USA). Measurements were taken at a temperature of 25 °C in a 10 mM tris buffer set at pH 7, assuming a continuous phase refractive index of 1.33 and a viscosity of 0.89 Pa s. In order to eliminate artefacts due to multiple scattering, all samples were diluted into the buffer and measured again. 

### 2.9. Emulsion Morphology

Micrographs were taken with an inverted Zeiss LSM 700 confocal microscope (Carl Zeiss, CZ Microscopy GmbH, Jena, Germany) in optical mode with a 20× lens. Prior to microscopic examination, 10 μL of 0.1mg mL^−1^ Nile Red and 10 μL of 0.1 mg mL^−1^ Nile Blue were added into each emulsion. A drop of each emulsion was placed on a welled glass slide and was covered with a coverslip before imaging.

## 3. Results and Discussion

**Figure 1:**
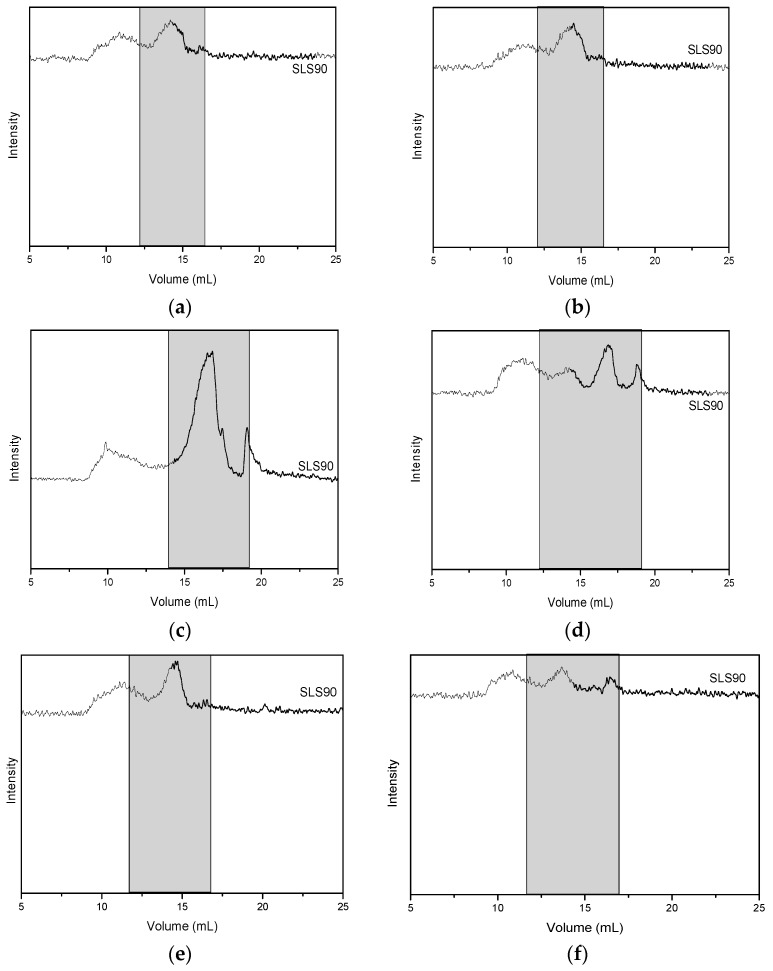
Size exclusion chromatograms of the extracts obtained from non-composted mixtures on the left column (**a**,**c**,**e**) and size exclusion chromatograms of the extracts obtained from partially composted mixtures on the right column (**b**,**d**,**f**). Top to bottom: Extracts obtained at pH 5 (**a**,**b**), pH 7 (**c**,**d**), and pH 9 (**e**,**f**). The greyed out areas highlight the regions of significant UV absorbance at 280 nm.

Figure 1 shows the results of size exclusion chromatography (SEC) for the products obtained by extractions at pH 5, pH 7, and pH 9 (henceforth to be called OC5, OC7, and OC9 respectively) from the initial material (prior to composting). The static light scattering (SLS) detector here records the angular intensity at 90°. At the visible wavelengths used, this signal was sensitive to the molecules of larger sizes (i.e., typically the ones eluting at lower volumes). The second detector (ultraviolet, UV, measuring here the absorbance at 280 nm) was expected to detect proteins based on the absorbance of their amino acids, namely near-UV absorbing Tyr, Trp, and, to a lesser extent, Phe and –S–S– bonds [13].

In samples OC5 (Figure 1a) and OC7 (Figure 1b), static light scattering (SLS) recorded two populations that scattered light at 90°: One eluting between 10 and 12 mL, and another between 12.5 and 15 mL. The second population absorbed strongly at 280 nm (see the chromatogram of the UV data). In sample OC9 (the one extracted at pH 9, Figure 1c), three scattering populations were distinct: One between 8 and 12 mL, corresponding to the elution times of dextran standards of MW > 1 MDa, a second one 14 and 17 (with a small peak at 17 mL), corresponding to the elution time of dextrans of several tens of kDa, and the third one at 18.5 mL, corresponding to smaller molecules (below 1 kDa). The populations corresponding to 14–17 mL, 17 mL, and 18.5 mL all absorbed at 280 nm. This suggested that the larger populations (those that elute prior to 15 mL) were composed of non-UV absorbing moieties; the only significant candidates for such molecules in plant-based food were polysaccharides. The second peaks should be attributed to proteinic structures, as they absorbed at 280 nm; the same applied for the smaller peak eluting immediately after the larger ones; while the smaller entities eluting at 18.5 mL in OC9 should be the breakdown products of proteins and other UV-absorbing molecules, such as phenolics.

Figure 1 also showed the evolution of these populations after the partial (one month) composting of the material: Samples OC5 showed a remarkable reduction in the size of its components (Figure 1d): The initial population of non-UV absorbing polysaccharides changed its shape from a bimodal peak to a monomodal one, with the entire area shifting to larger elution times. The protein peak, initially between 12.5 and 15 mL (see uncomposted samples in Figure 1), broke down into a smaller peak of the same size, and a much larger one UV-absorbing peak eluting at 17 mL, suggesting the break-up of the protein population during composting. A further new peak appeared at much higher elution times (19 mL), corresponding to the elution time of standard of MW below 1000 Da; that is, this peak was the individual amino acids or oligopeptides deriving from the cleavage of the initial proteinic population. While such changes are not readily apparent in OC7 (Figure 1e) and OC9 (Figure 1f), where the main populations appeared to remain largely unaffected by the short composting process. Overall, OC5 showed promise as the partially-completed composting process started cleaving the macromolecules into smaller ones, of possibly better emulsifying characteristics.

The zeta potential of OC5 was measured to be −9.3 ± 1.0 mV, while that of OC7 was measured to be −26.8 ± 1.0 mV; the reader should be reminded that all zeta potential measurements were taken at pH 7, using samples extracted at different pH values (OC5, OC7, OC9); so their different zeta potentials reflected real compositional differences. That suggested that entities of higher density in ionizable moieties (e.g., carboxyls) were extracted at pH 7 and pH 9 (as compared to pH 5); this could explain the smaller content of degradation products (or products of lower MW) in OC7 and C9 as compared to OC5: For a molecule to be extracted from a plant matrix, the force between the hydrating water and the molecule under extraction should be larger than the forces acting between that molecule and the plant matrix [14]. At pH 7 or 9, the charges were higher than at pH 5 (as at pH 5, most proteins were closer to their pI). So electrostatic interactions between the plant matrix and the molecules under extraction were weaker, facilitating their extraction.

**Figure 2:**
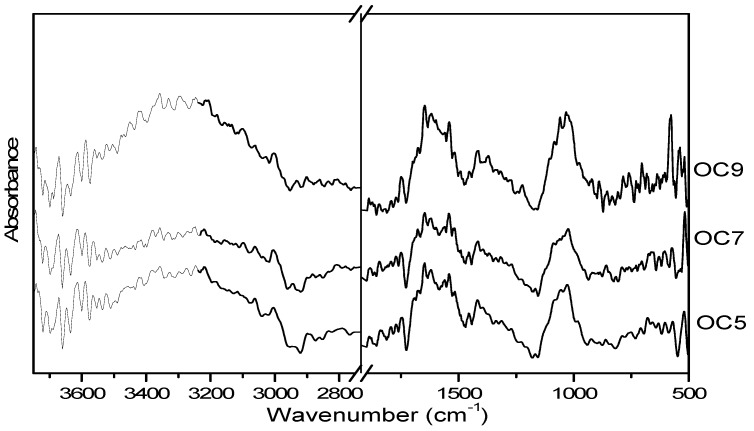
Fourier-transform infra-red spectroscopy (FTIR) spectra of the partially composted waste extracts obtained at pH 5 (OC5), at pH 7 (OC7), and at pH 9 (OC9).

Figure 2 shows the Fourier-transform infra-red (FTIR) spectra of the three materials, as obtained by direct application of the samples on an ATR module. No differences were expected to be found between proteins and their broken down products, as they contained essentially the same vibrating units. However, the FTIR examination could yield data on the chemical identity of the complex mixtures that form these extracts. The major peaks at 1020–1090 cm^−1^ were typical of polysaccharidic entities [15]). The peak groups from 1530 to 1630 cm^−1^ and 1280 to 1450 cm^−1^ were due to proteins and other peptidic entities (i.e., proteins, oligopeptides, and individual aminoacids), attributable to C=O stretching (amide I region) and to N–H bending (amide II region) [16]. Both polysaccharides and proteins have been detected as separate populations in the SEC (Figure 1). A mild shoulder existed at 1540 cm^−1^, which was more pronounced in samples OC7 and OC9. This was due to carboxylate bending and correlated well with the higher absolute values of (negative) zeta potential of these two samples. The shoulders around 3040 and 3560 cm^−1^ were typical stretching vibrations of O–H units (present in all sugars, peptides, and residual water) [17].

**Figure 3:**
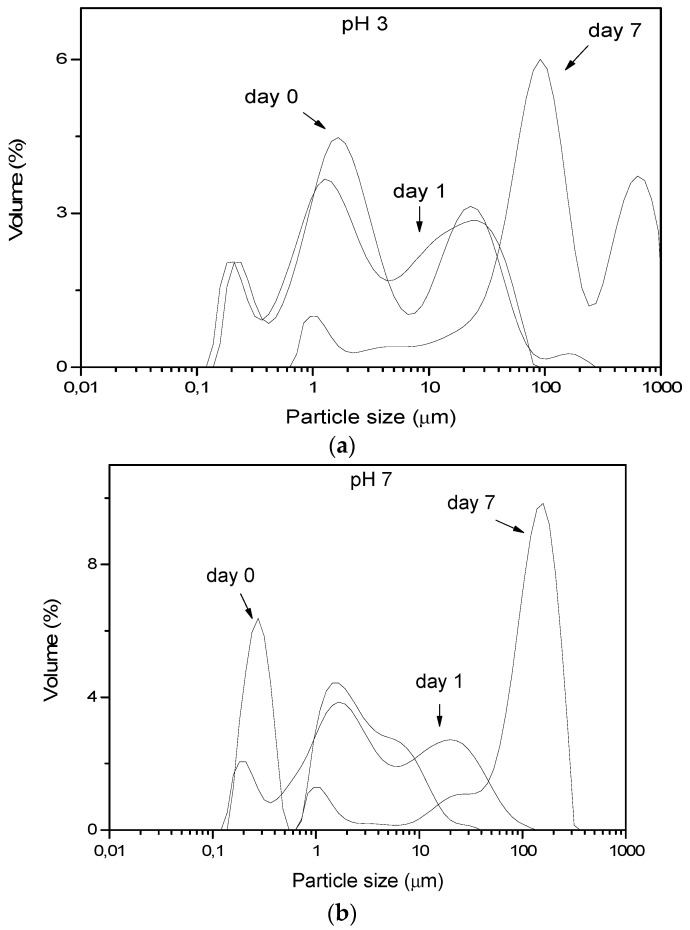
Size distribution of the particles in miglyol-in-water emulsions prepared usingas emulsifier the extract obtained from partially composted olive waste+leaf mixtures extracted at pH 5 (OC5). The emulsions pH is 3 (**a**) and 7 (**b**).

The extract obtained at pH 5 (OC5) has been shown in Figure 1 to comprise of more low-MW components, as the composting process has begun to affect the sample in question. So OC5 has been used as an emulsifier for oil–in–water emulsions set at pH 3 and pH 7 as to simulate neutral and acidic soft foods, respectively. Figure 3 shows the size distributions of the produced droplets and flocs at these two pH values, as they evolved over one week of storage. The top part shows the droplet size distributions for emulsions at pH 3 (Figure 3a). The initial droplets were centered around two populations, around 0.2 and 1 μm, and a population of larger droplets or flocs also existed around 20 μm. These were smaller than the droplets obtained from uncomposted olive extract [11], while they were also smaller than the droplets obtained from emulsifiers from winery extract [10], quince seed [9], and okra-derived emulsifiers [18]. The droplet size distributions remained stable after 24 h, although over 7 days a small 1 μm peak remained, most of the oil existed at entities of 100 μm or above. In that aspect, running the same samples after insertion of 1 g dL^−1^ sodium dodecyl sulphate (SDS) did not change the droplet size distribution (results not shown); that is, displacement of the interfacial layer by SDS does not result in the breaking-up of any flocs; so the larger-sized peaks were most probably individual droplets produced either by coalescence or by Ostwald ripening. The latter mechanism of destabilization has also been clearly demonstrated as the main threat to the stability of emulsions prepared at pH 3 by okra extracts [18]. That suggests that the emulsifiers obtained by OC5 are promising for use in acidic foods, given that the product shelf-life will be restricted.

**Figure 4:**
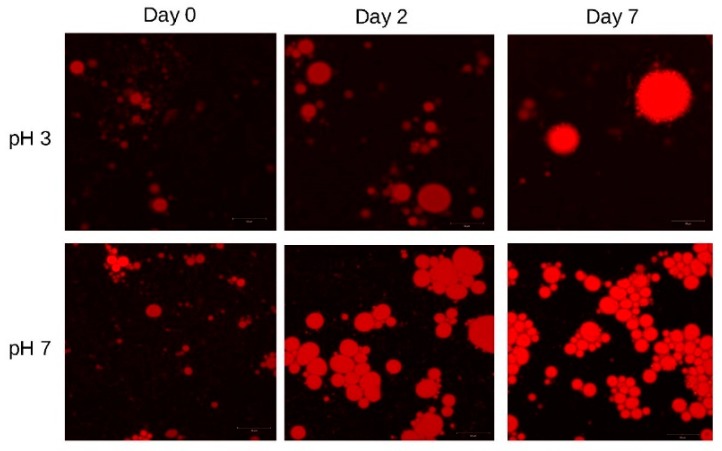
Confocal micrographs of miglyol-in-water emulsions prepared using the extract obtained from partially composted olive waste+leaf mixtures at extraction pH 5 (OC5). The pH values of the emulsions are 3 (top) and 7 (bottom); migrographs are taken (from left to right) upon preparation; after two days of storage; and after seven days of storage.

The image obtained at pH 7 (Figure 3b) was different by means of the fast increase of the droplet size within 24 h from preparation. This is reminiscent of the inability of okra extracts to emulsify at pH 7 [18], while non-composted olive mill waste is shown to produce stable emulsions, possibly due to the Pickering action of the larger, non-hydrolyzed macromolecules of the non-composted sample [11]. Micrographic examination of the systems under study (Figure 4) showed clearly the increase in droplet sizes over time; while at pH 3 a few droplets grew at the expense of the smaller ones, at pH 7 the large droplets were dominant, in agreement with the laser particle sizing data of Figure 3. The increase of the larger droplets at the expense of the smaller ones was very clearly depicted as a large number of small droplets flocculated with single large droplets at pH 3 (upper right micrographs, pH 3 at 7 days). Similar observations could also be made for the large droplets at pH 7 (lower part micrographs). A rightward shift of the droplet size distribution, coupled with the growing was the size of the larger droplets at the expense of the smaller ones, was indicative of Ostwald ripening, rather than coalescence [14]. This means that a strong mechanical layer, capable of protecting against droplets merging/coalescing, was indeed formed by the novel emulsifiers; in order to control; the stability of these emulsions, one should seek to control parameters which influence Ostwald ripening rather than coalescence, such as the surface elasticity, the solubility of the oil into water, the surface tension and the viscosity.

These observations make an interesting and promising opening for further investigation of the potential of non-complete composting in producing high added-value products. The extraction of hydrocolloids, emulsifiers, or other polyphenolics from the partially-composted agricultural waste can provide a novel, fast, and economic way of valorization of the extensive waste of the agricultural industry. This is an unexplored path that is worth further investigation.

## 4. Conclusions

Partial composting of olive processing solid wastes can yield emulsifiers that are capable of absorbing onto the oil–water interfaces of acidic emulsions and stabilizing against coalescence; they are less successful in stabilizing against Ostwald ripening. The optimum extraction pH is 5. The ability of these emulsifiers to stabilize the emulsions for some days is due to the presence of the break-down products the solid waste’s proteinic and polysaccharidic components that are produced during composting. Extractions at higher pH values do not yield such break-down products, as electrostatic attractions keep the products attached to their plant matrix. Overall, this work serves as a demonstration of the capacity of restricted composting to produce high added-value products.
